# Effects of Social Distancing Measures during the First Epidemic Wave of Severe Acute Respiratory Syndrome Infection, Greece

**DOI:** 10.3201/eid2702.203412

**Published:** 2021-02

**Authors:** Vana Sypsa, Sotirios Roussos, Dimitrios Paraskevis, Theodore Lytras, Sotirios Tsiodras, Angelos Hatzakis

**Affiliations:** National and Kapodistrian University of Athens, Athens, Greece (V. Sypsa, S. Roussos, D. Paraskevis, S. Tsiodras, A. Hatzakis);; National Public Health Organization, Athens (T. Lytras);; European University Cyprus, Nicosia, Cyprus (T. Lytras)

**Keywords:** respiratory infections, severe acute respiratory syndrome coronavirus 2, SARS-CoV-2, SARS, COVID-19, coronavirus disease, zoonoses, viruses, coronavirus, epidemiology, basic reproduction number, social distancing, models, Greece

## Abstract

Greece imposed a nationwide lockdown in March 2020 to mitigate transmission of severe acute respiratory syndrome coronavirus 2 during the first epidemic wave. We conducted a survey on age-specific social contact patterns to assess effects of physical distancing measures and used a susceptible-exposed-infectious-recovered model to simulate the epidemic. Because multiple distancing measures were implemented simultaneously, we assessed their overall effects and the contribution of each measure. Before measures were implemented, the estimated basic reproduction number (R_0_) was 2.38 (95% CI 2.01–2.80). During lockdown, daily contacts decreased by 86.9% and R_0_ decreased by 81.0% (95% credible interval [CrI] 71.8%–86.0%); each distancing measure decreased R_0 _by 10%–24%. By April 26, the attack rate in Greece was 0.12% (95% CrI 0.06%–0.26%), one of the lowest in Europe, and the infection fatality ratio was 1.12% (95% CrI 0.55%–2.31%). Multiple social distancing measures contained the first epidemic wave in Greece.

Coronavirus disease (COVID-19), caused by severe acute respiratory syndrome coronavirus 2 (SARS-CoV-2), emerged in China in December 2019 (*1*) and by September 14, 2020, had spread worldwide, causing >28.6 million cases and >917,000 deaths (*2*). To suppress the epidemic curve, public health authorities needed to use the strongest possible mitigation strategies until effective therapies and vaccines are available. Central mitigation strategies include nonpharmaceutical interventions, such as travel-related restrictions, case-based, and social distancing interventions. Social distancing aims to decrease social contacts and reduce transmission (*3*).

In Greece, the first COVID-19 case was reported on February 26, 2020 (*4*). Soon after, several social distancing, travel-related, and case-based interventions were implemented. A nationwide lockdown restricting all nonessential movement throughout the country began on March 23 (Figure 1). By the end of April, the first epidemic wave had waned, and withdrawal of physical distancing interventions became a social priority. 

**Figure 1:**
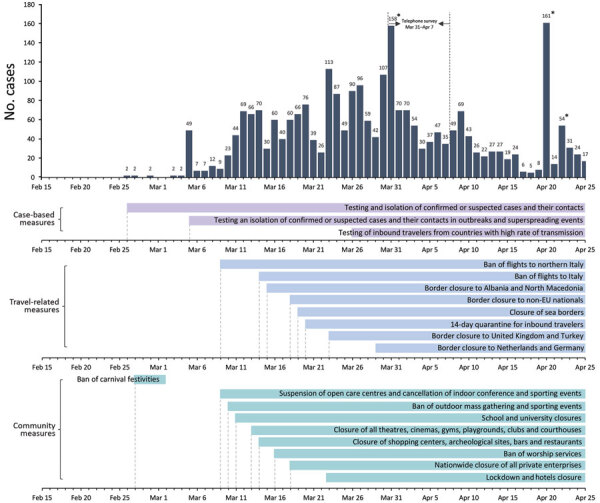
Daily number of coronavirus disease cases by date of sampling for laboratory testing (25) and timeline of key measures, Greece. Dates of telephone survey are indicated. Asterisks indicate spikes in the number of diagnosed cases at the end of March and late April that correspond to clusters of cases in 3 settings: a ship, a refugee camp, and a clinic. EU, European Union.

Despite an ongoing severe financial crisis and an older population, Greece has been noted as an example of a country with successful response against COVID-19 (*5*). However, given the resurgence of cases in Greece and other countries, careful consideration and close monitoring are needed to inform strategies for resuming and maintaining social and economic activities.

We describe a survey implemented during lockdown in Greece and assess the effects of physical distancing measures on contact behavior. We used these data and mathematical modeling to obtain estimates for the first epidemic wave in the country, during February–April 2020, to assess the effects of all social distancing measures, and to assess the relative contribution of each measure towards the control of COVID-19.

## Materials and Methods

### Social Contacts Survey

We conducted a phone survey during March 31–April 7, 2020, to estimate the number of social contacts and age mixing of the population on a weekday during the lockdown and on the same day of the week before the pandemic, during mid-January 2020, by using contact diaries (Appendix Figure 1). Participants provided oral informed consent. We defined contact as either skin-to-skin contact or a 2-way conversation with >3 words spoken in the physical presence of another person (*6*). For each contact, we recorded information on the contact person’s age and location of the contact, such as home, school, workplace, transportation, leisure, or other. We planned to recruit 600 participants of all ages residing in Athens by using proportional quota sampling and oversampling among persons 0–17 years of age. 

We estimated the average number of contacts for the prepandemic and lockdown periods. We defined 6 age groups to build age-specific contact matrices, adjusting for the age distribution of the population of Greece, by using socialmixr in R software (R Foundation for Statistical Computing, https://www.r-project.org).

### Estimating the Course of the First Epidemic Wave and Assessing Effects of Social Distancing

To estimate the course of the epidemic, we first estimated the basic reproduction number (R_0_), the average number of secondary cases 1 case would produce in a completely susceptible population in the absence of control measures. Then, we used social contacts matrices to assess the effects of physical distancing measures on R_0_. Finally, we simulated the course of the epidemic using a susceptible-exposed-infectious-recovered (SEIR) model.

#### Estimating R_0_

We estimated R_0_ based on the number of confirmed cases with infection onset dates before the first social distancing measures were adopted, up to March 9, and accounted for imported cases. We used a maximum-likelihood method to obtain the R_0_ and 95% CI, assuming that the serial interval distribution is known (*7*). We used the daily number of cases by date of symptom onset and inferred infection dates assuming an average incubation period of 5 days (*8*,*9*). We assumed a gamma distributed serial interval with a mean of 6.67 (SD 4.85) days, in accordance with other studies (*10*,*11*; D. Cereda et al., unpub. data, https://arxiv.org/abs/2003.09320). As a sensitivity analysis, we estimated R_0_ assuming a shorter serial interval of 4.7 days (Appendix) (*12*).

#### Assessing Effects of Social Distancing on R_0_

Primary social distancing measures implemented in Greece began on March 11. These measures and the dates implemented were closing all educational establishments on March 11; theatres, courthouses, cinemas, gyms, playgrounds, and nightclubs on March 13; shopping centers, cafes, restaurants, bars, museums, and archaeological sites on March 14; suspending services in churches on March 16; closing all private enterprises, with some exceptions, on March 18; and, finally, restricting all nonessential movement throughout the country on March 23 (Figure 1; Appendix Table 1).

We assessed the effects of these measures on R_0_ through the social contact matrices obtained before and during lockdown, as used in other studies (*13*,*14*). For respiratory-spread infectious agents, R_0_ is a function of the age-specific number of daily contacts, the probability that a single contact leads to transmission, and the total duration of infectiousness; thus, R_0_ is proportional to the dominant eigenvalue of the social contact matrix (*15*). If the other 2 parameters did not change before and during social distancing measures, the relative reduction, δ, in R_0_ is equivalent to the reduction in the dominant eigenvalue of the contact matrices obtained for the 2 periods (Appendix) (*14*,*16*). To account for a lower susceptibility for children than for adults, we introduced an age-dependent proportionality factor, *s_i_*, measuring susceptibility to infection of persons in age group *i*, as in other studies (*13*,*17*). We performed the analysis using a conservative estimate for *s_i_*, and considered the susceptibility among persons 0–17 years of age to be 0.34 compared with persons >18 years of age (Appendix Table 2) (*13*).

We estimated the relative reduction in R_0_ in 2 periods: the period of initial measures until the day before lockdown (March 11–22), which included closure of schools, entertainment venues, and shops (reduction δ_1_); and the period of lockdown (March 23–April 26) (reduction δ_2_). Because we did not assess social contacts during the period of initial measures, we created a synthetic contact matrix by assuming no school contacts because of school closures, and a reduction in leisure and work contacts (*18*–*20*) (Appendix). To assess uncertainty, we performed a nonparametric bootstrap on contact data by participant to estimate the mean and 95% credible interval (95% CrI) of δ_1_ and δ_2_ (n = 1,000 bootstrap samples).

### Simulating the Epidemic in Greece

**Figure 2:**
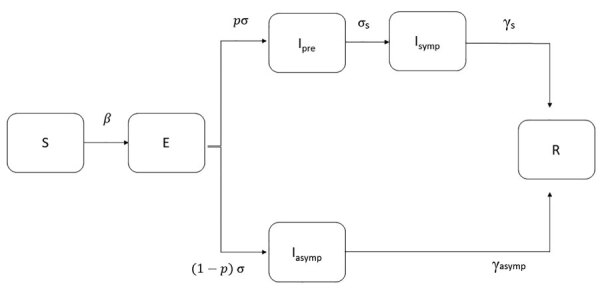
Modified susceptible-exposed-infectious-recovered (SEIR) model used to estimate the course of the first epidemic wave of coronavirus disease, Greece. Cases are classified into susceptible (S), exposed (E), infectious (I, which is divided into 3 conditions: I_pre_, before developing symptoms, I_symp_ for clinically ill, or I_asymp_ for true asymptomatic), and recovered (R). We assumed that a proportion (*p*) of exposed cases will develop symptoms and that infectiousness can occur before the onset of symptoms. β is the rate at which persons become infected and move to E; exposed individuals become infectious at a rate *σ* and presymptomatic infectious cases develop symptoms at a rate *σ_s_*; γ_asymp_ is the rate of recovery for asymptomatic persons; γ_s_ is the rate of recovery for symptomatic persons.

We used a SEIR model to simulate the outbreak from the beginning of local transmission until April 26, 2020, the day before the originally planned date to ease lockdown measures. Susceptible persons (S) become infected at a rate β and move to the exposed state (E) as infected but not infectious. Exposed persons become infectious at a rate σ, and a proportion *p* will eventually develop symptoms (*p* = 80%) (*21*). To account for asymptomatic transmission during the incubation period, we introduce a compartment for infectious presymptomatic persons (I_pre_). I_pre_ cases become symptomatic infectious (I_symp_) cases at a rate of σ_s_. We assumed that infectiousness can occur 1.5 days before the onset of symptoms (*22*–*24*). The remainder (*1 – p*) will be true asymptomatic or subclinical cases (I_asymp_)_._ We assumed that the infectiousness of subclinical cases relative to symptomatic cases was *q* = 50% (*24*). Symptomatic cases recover (R) at a rate of γ_s_, and asymptomatic cases recover (R) at a rate of γ_asymp_ (Table 1; Figure 2; Appendix).

**Table 1:** Parameters of the susceptible-exposed-infectious-recovered model used to assess effects of social distancing measures during the first epidemic wave of coronavirus disease, Greece

Epidemiologic parameters	Value	Comments and references
R_0_ (95% CI)	2.38 (2.01–2.80)	Estimated from data on the number of confirmed cases in Greece by accounting for imported cases and assuming gamma distributed serial interval with mean 6.67 days (SD 4.88 days) (D. Cereda et al., unpub. data, https://arxiv.org/abs/2003.09320) and aligned with other studies (*10**,**11*)
Latent period (1/σ)	3.5 days	Based on an average incubation time of ≈5 days (*8**,**9*) and assuming that infectiousness starts 1.5 days prior to the symptom onset (*22**–**24*)
Percentage (*p*) infected cases developing symptoms	80	From K. Mizumoto et al. (*21*), the estimated proportion of true asymptomatic cases was 20.6% assuming a mean incubation period of 5.5 days
Symptomatic cases		
Length of infectiousness before symptoms, d (1/σ_s_)	1.5	(*22**–**24*)
Duration of infectious period from development of symptoms to recovery, d (1/γ_s_)	4.5	To obtain a serial interval of ≈6 days (*8**,**9*)
True asymptomatic cases		
Infectiousness (*q*) of asymptomatic vs. symptomatic persons, %	50	(*24*)
Duration of infectious period until recovery (1/γ_asymp_)	6 days	The same duration of infectiousness as for symptomatic cases = 1/σ_s_ + 1/γ_s_

We derived the transmission rate β from R_0_ and parameters related to the duration of infectiousness (Appendix). We incorporated uncertainty in R_0_ by drawing values uniformly from the estimated 95% CI (2.01–2.80). We modeled the effect of measures by multiplying β by the parameters δ_1_ and δ_2_; in which δ_1_ corresponds to the reduction of R_0_ in the period of initial social distancing measures, where δ_1_ was drawn from a normal distribution with a mean of 42.7% (SD 1.7%); and δ_2_ corresponds to the reduction of R_0_ during lockdown, for which δ_2_ was drawn from a normal distribution of 81.0% (SD 1.6%) estimated from the bootstrap on the contact data. To account for the uncertainty in R_0_, δ_1_, and δ_2_, we performed 1,000 simulations of the model and obtained median estimates and 95% CrIs.

We obtained the infection fatality ratio (IFR) and the cumulative proportion of critically ill patients by dividing the reported number of deaths and of critically ill patients (*25*) by the total number of cases predicted by the model. We used a lag of 18 days for deaths and 14 days for critically ill patients based on unpublished data on hospitalized patients from the National Public Health Organization in Greece. To validate our findings, we used a reverse approach; we applied a published estimate of the IFR (*26*) to the number of infections predicted by the model and compared the resulting cumulative and daily number of deaths to the observed deaths (Appendix Table 3).

### Effects of Social Distancing Interventions

Because multiple social distancing measures were implemented simultaneously, to delineate the effects of each measure on R_0_, we used information from the contacts reported on a regular weekday in January 2020 and mimicked the impact of each intervention by excluding or reducing subsets of corresponding social contacts (*16*,*17*,*19*,*20*) (Appendix). We also assessed scenarios with less disruptive social distancing measures (Appendix). In addition, we evaluated the increase in effective reproduction number (R_t_) for varying levels of infection control measures (hand hygiene, use of facemasks, and maintaining distance >1.5 m) when social distancing measures are partially lifted after lockdown (Appendix).

## Results

### Social Contacts before and during Lockdown

In total, 602 persons provided contact diaries and reported 12,463 contacts before the pandemic and 1,743 during lockdown (Table 2). The mean daily number of contacts declined from 20.7 before to 2.9 during lockdown; when adjusted for the age distribution of the population, the reduction was 19.9 before and 2.6 during lockdown (86.9%).

**Table 2:** Number of contacts on a weekday during lockdown, March 31–April 7, 2020, and on the corresponding day in January 2020 before the coronavirus disease epidemic in Athens, Greece

Covariate	Mid-January 2020				During lockdown		Reduction of reported contacts, %
	Participants, no. (%)	No. (%)	Mean (95% CI)		No. (%)	Mean (95% CI)	
Overall	602 (100.0)	12,463 (100.0)	20.7 (18.9–22.5)		1,743 (100.0)	2.9 (2.6–3.2)	86.0*
Sex							
M	295 (49.0)	6,218 (49.9)	21.1 (18.3–23.9)		934 (53.6)	3.2 (2.7–3.6)	85.0
F	307 (51.0)	6,245 (50.1)	20.3 (18.0–22.7)		809 (46.4)	2.6 (2.2–3.1)	87.1
Age, y							
0–4	20 (3.3)	386 (3.1)	19.3 (12.8–25.8)		53 (3.0)	2.7 (2.2–3.1)	86.3
5–11	58 (9.6)	2,020 (16.2)	34.8 (29.1–40.6)		168 (9.6)	2.9 (2.6–3.2)	91.7
12–17	83 (13.8)	2,758 (22.1)	33.2 (28.4–38.1)		275 (15.8)	3.3 (2.3–4.3)	90.0
18–29	74 (12.3)	1,316 (10.6)	17.8 (14.4–21.1)		361 (20.7)	4.9 (3.1–6.7)	72.6
30–64	209 (34.7)	4,852 (38.9)	23.2 (19.5–26.9)		529 (30.4)	2.5 (2.2–2.9)	89.1
>65	158 (26.3)	1,131 (9.1)	7.2 (5.4–8.9)		357 (20.5)	2.3 (1.8–2.7)	68.4

*The reduction in the reported contacts becomes 86.9% after adjusting for the age distribution of the population of Greece.

**Figure 3:**
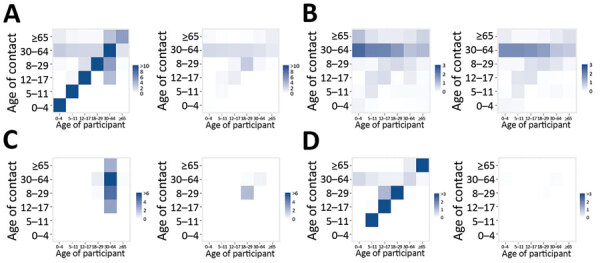
Side-by-side comparisons of age-specific contact matrices in Greece before the coronavirus disease pandemic (January 2020; left) and during lockdown (April 2020; right). A) All contacts; B) contacts at home; C) contacts at work; and D) contacts during leisure activities. Each cell represents the average daily number of reported contacts, stratified by the age group of the participants and their corresponding contacts. In panel A, the diagonal of the contact matrix corresponds to contacts between persons in the same age group, the bottom left corner of the matrix corresponds to contacts between school-age children, and the central part corresponds to contacts mainly in the work environment.

We noted a change in age-mixing patterns in the contact matrices (Figure 3, panel A). In the prepandemic period, the diagonal of the contact matrix depicts the assortativity by age; participants tended to associate more with people of similar age (Figure 3, panel A). When social distancing measures were put into effect, the assortativity by age disappeared and contacts occurred mainly between household members (Figure 3, panels B–D).

### R_0_ and Effects of Social Distancing Measures

**Figure 4:**
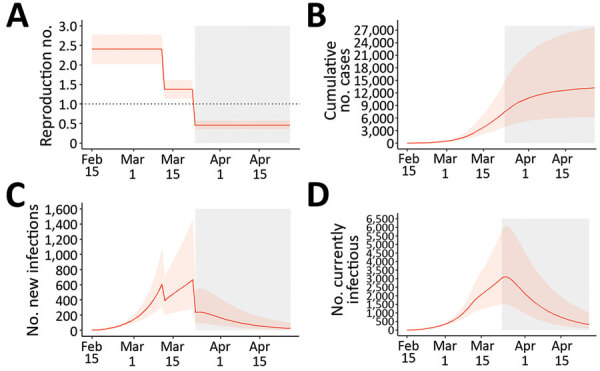
The first wave of the coronavirus disease epidemic in Greece (February 15–April 26, 2020), estimated from 1,000 susceptible-exposed-infectious-recovered (SEIR) model simulations. A) Effective reproduction number; B) cumulative number of cases; C) new infections; and D) number of infectious persons by date. Orange lines represent the median estimates, and the light orange shaded areas indicate 95% credible intervals. Gray areas indicate the period of restrictions of all nonessential movement in the country (i.e., lockdown).

Before lockdown, the estimated R_0_ was 2.38 (95% CI 2.01–2.80). During the first period of social distancing measures, in which schools, entertainment venues, and shops were closed, R_0_ was estimated to decrease by 42.7% (95% CrI 34.9%–51.3%); under lockdown, R_0_ decreased by 81.0% (95% CrI 71.7%–86.1%). Thus, the cumulative measures implemented during lockdown would have reduced R_0_ to <1.0 even if the initial R_0_ had been as high as 5.3 (95% CrI 3.5–7.2). Estimated R_t_ was 1.13 (95% CrI 1.38–1.61) during the period of the initial measures but was 0.46 (95% CrI 0.35–0.57) during lockdown (Figure 4, panel A).

### Contribution of Each Social Distancing Measure

**Figure 5:**
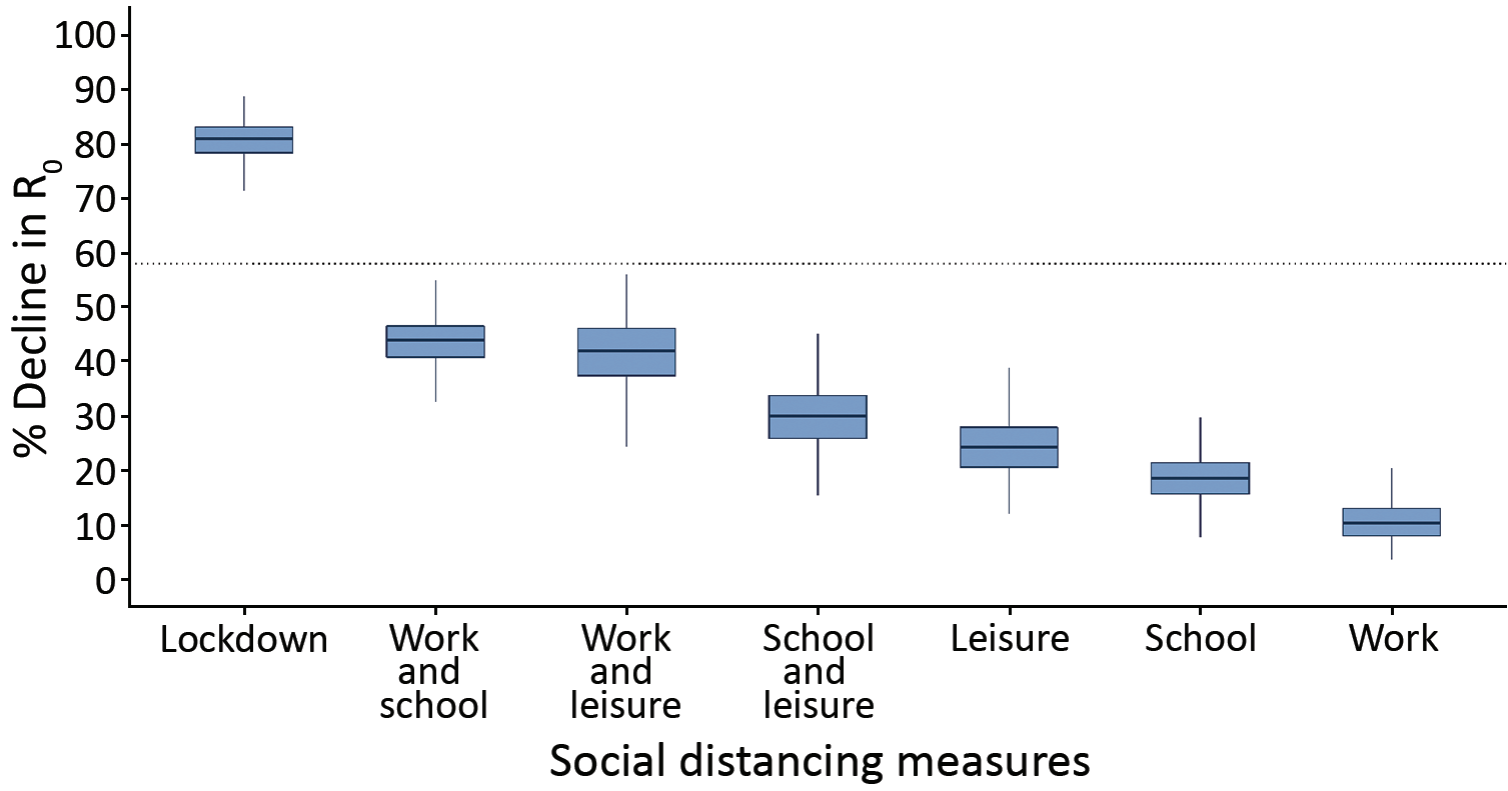
The percentage of decline of R_0_ associated with multiple social distancing measures during coronavirus disease lockdown in Greece and the relative contribution of each measure or combination of measures implemented. Boxplots demonstrate distribution of the estimated percent decline from nonparametric bootstrap on the social contacts data based on 1,000 bootstrap samples. R_0_ reduction during lockdown was obtained by comparing social contacts data collected for April 2020 versus January 2020. The other estimates were derived by using the information from contact diaries in January 2020 corresponding to a regular school or work day and excluding or reducing subsets of social contacts at school, work, home, and leisure activities, based on observations during lockdown. Because contact with a particular person can take place in multiple settings, we assigned contacts at multiple locations to a single location by using the following hierarchical order: home, work, school, leisure activities, transportation, and other locations. Dotted line indicates the minimum reduction needed to bring R_0_ from 2.38 to <1. Box top and bottom lines indicate 25th and 75th percentiles; horizontal lines within boxes indicate medians; whiskers indicate 25th/75th percentile plus 1.5 times the interquartile range. R_0_, basic reproduction number.

We assessed the effect of each measure separately and in combinations (Figure 5). During lockdown, the estimated reduction in R_0_ attributed to each measure was 10.3% (95% CrI 5.2%–20.3%) for the decline in work contacts, 18.5% (95% CrI 10.7%–26.3%) for school closures, and 24.1% (95% CrI 14.8%–34.3%) for the decline in leisure activity contacts. Thus, each measure separately would have reduced R_0_ to <1.0 if the initial R_0_ had been as high as 1.11 for the decline in work contacts, 1.23 for school closures, and 1.32 for the decline in leisure activity contacts. A combination of measures could be effective if the initial R_0_ had been as high as 1.78 for interventions reducing work and school contacts, 1.72 for reducing work and leisure contacts, and 1.43 for reducing school and leisure contacts.

We assessed alternative scenarios with less disruptive social distancing measures. A 50% reduction in school contacts, such as smaller class sizes; 20% in work contacts, such as teleworking for part of the population or rotating weekly schedules in which employees telework some days and work onsite other days; and 20% in leisure activities could reduce R_0_ to <1.0 for initial levels as high as 1.32 (95% CrI 1.27–1.38). An even larger decline in leisure activities (50%) could successfully reduce an initial R_0_ as high as 1.48 (95% CrI 1.35–1.62). 

**Figure 6:**
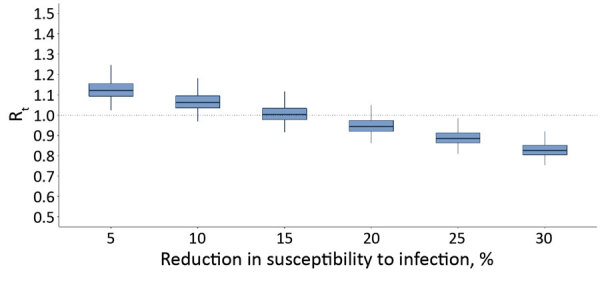
Estimated R_t_ after the partial lifting of social distancing measures at the end of the first coronavirus disease epidemic wave in Greece for varying effectiveness levels of infection control measures, such as hand hygiene, use of masks, maintaining social distances, in reducing susceptibility to infection. R_t_ during lockdown was 0.46. For the partial lifting of measures, we hypothesized a scenario in which contacts at work and school contacts will return to 50% lower than pre-epidemic levels and leisure activities will return to 60% lower than pre-epidemic levels. Dotted line indicates the threshold of R_t_ = 1. Boxplots of the distribution of the estimated Rt from nonparametric bootstrap on the social contacts data based on 1,000 bootstrap samples. Box top and bottom lines indicate 25th and 75th percentiles; horizontal lines within boxes indicate medians; whiskers indicate 25th/75th percentile plus 1.5 times the interquartile range. R_t_, effective reproduction number.

Finally, we assessed the increase in R_t_ when measures were partially lifted after lockdown*.* To mimic the measures implemented after lockdown in Greece, we assumed that contacts at work would return to levels 50% lower than pre-pandemic, school to 50%, and leisure to 60%. For instance, class sizes were reduced 50% when schools reopened in May. Under this scenario, R_t_ would remain <1.0 assuming >20% reduction in susceptibility as a result of infection control measures, including hand hygiene, use of face masks, and maintaining physical distances >1.5 meters (Figure 6). Under milder social distancing measures, infection control policies would need to be much more effective (Appendix Figure 2).

### Model Predictions on the Epidemic during February 15–April 26

By April 26, 2020, Greece had 2,517 diagnosed COVID-19 cases, 23.0% of which were imported, and 134 deaths (Figure 1) (*25*). The corresponding naive case-fatality ratio (CFR) was 5.3%. Based on our SEIR model, the cumulative number of infections during February 15–April 26 would be 13,189 (95% CrI 6,206–27,700) (Figure 4, panel B), which corresponds to an attack rate (AR) of 0.12% (95% CrI 0.06%–0.26%). The estimated case ascertainment rate was 19.1% (95% CrI 9.1%–40.6%). By the end of April, 25 (95% CrI 6–97) new infections per day and 329 (95% CrI 97–1,027) total infectious cases were estimated (Figure 4, panels C, D).

On the basis of the number of deaths and critically ill patients reported in Greece by April 26, and using the number of infections obtained from the model as denominator, we estimated the IFR to be 1.12% (95% CrI 0.55%–2.31%) and the cumulative proportion of critically ill patients to be 1.55% (95% CrI 0.75%–3.22%). As a validation, we estimated the number of deaths by applying a published age-adjusted estimated IFR to the number of infections predicted by the model (Appendix Table 3). The predicted number of deaths was 137 (95% CrI 66–279) compared with the reported number of 134 deaths (Appendix Figure 3). As a sensitivity analysis, we simulated the epidemic and calculated IFR and AR assuming a shorter mean serial interval of 4.7 days. We obtained similar results for the AR and the IFR as when the serial interval was 6.67 days (Appendix Figure 4).

## Discussion

Greece and other countries managed to successfully slow the first wave of the SARS-CoV-2 epidemic early in 2020. Assessing the burden of infection and death in the population and quantifying the effects of social distancing was necessary because the stringent measures taken had major economic costs and restricted individual freedom. In addition, several countries, including Greece, began seeing COVID-19 cases increase after resuming economic activities and travel, indicating the need to reimplement some types of location-specific physical distancing measures.

We assessed the effects of social distancing by using a social contacts survey to directly measure participants’ contact patterns during lockdown in a sample including children. To our knowledge, only 2 other diary-based social contacts surveys have been implemented during COVID-19 lockdown, 1 in China (*13*) and 1 in the United Kingdom (*14*); only the study from China included children. Our study had common findings with the other 2: a large reduction in the number of contacts, 86.9% in Greece, 86.4%–90.3% in China, and 73.1% in United Kingdom; and assortativity by age (i.e., contacts between people of the same age group) disappeared during lockdown and contacts were mainly among household members. Other studies have assessed the impact of social distancing indirectly by using contact data from prepandemic periods and assuming that interventions reduce social mixing in different contexts (*18*,*20*,*27*).

We estimated that R_0_ declined by 81% and reached 0.46 during lockdown. This finding agrees with findings from a study pooling information from 11 countries in Europe, which also reported an 81% reduction in R_0_ (*28*) and with estimates from China (*3*,*29*), the United Kingdom (76.2%; *14*), and France (77%; *30*). In our analysis, we assumed lower susceptibility among children because of support from a growing body of evidence (*13*,*17*,*31*–*33*; K. Mizumoto et al., unpub. data, https://doi.org/10.1101/2020.03.09.20033142).

We further attempted to delineate the effects of each measure. For example, many countries, including Greece, instituted large-scale or national school closures (*34*). We estimated that each measure alone could reduce an R_0_ of ≈1.1–1.3 to <1.0. Only multiple social distancing measures would be effective for reducing an R_0_ at the initial level (2.38) observed in Greece. The finding concerning an 18.5% reduction in R_0_ related to school closures agrees with recent studies suggesting that this measure likely is much less effective for COVID-19 than for influenza-like infections (*17*,*28*). Concerning the course of the epidemic after lockdown, moderately relaxing social distancing could be safe if ongoing infection control strategies are adopted; milder social distancing measures would demand stricter infection control policies.

By May 18, 2020, Greece had one of the lowest reported COVID-19 death rates in Europe, 15.2 deaths/1 million population (*35*) (Appendix Table 4). Our IFR estimate of 1.12% was similar to that anticipated for the population of Greece based on a published estimate adjusting for demography (*26*). In addition, the estimated AR of 0.12% (95% CrI 0.06%–0.26%) was one of the lowest in Europe (*28*,*36*). Other researchers have applied back calculation of infections from reported deaths (*28*), and the resulting infection AR was almost identical (0.13%) (*36*). Our estimate is further confirmed by a serosurvey in residual serum samples that identified 0.25% (95% CI 0.02%–0.50%) seroprevalence in Greece in April 2020 (*37*). The number of infectious cases subsided considerably towards the end of April; however, even during this period with low transmission levels, 2 local outbreaks were identified, 1 in a refugee camp and 1 in a private healthcare unit, thus increasing the number of diagnosed cases in the respective days (Figure 1). An increasing number of reports around the world suggest the significance of superspreading events (*38*–*41*), and caution should be exercised to prevent or recognize these events early.

The first limitation of our study was that, due to the absence of prepandemic data on social contacts, we asked respondents to report their contacts ≈2 months prior to the survey to ensure reports were not affected by increased awareness of the pandemic. Recall bias might be observed, although to what direction is not clear. A general limitation in contact diaries is that participants record a fraction of their contacts (*42*). However, biases in participant recall are difficult to quantify, especially for those with many contacts in different settings. For example, short-lived contacts and work contacts are more likely to be underreported (*42*). Thus, recall bias could be different among children and adults and in various settings. In addition, underreporting might have occurred before and during lockdown because of many social contacts before the pandemic or because participants were afraid to disclose contacts during lockdown. Second, the survey was conducted in a sample from the Athens metropolitan area and not from the whole country. However, no consistent relationship has been found between social contacts and urbanization (*43*). In addition, most (79%) of the population of Greece lives in urban areas, and Athens accounts for 35% of the population. Furthermore, the observed reduction of social contacts during lockdown was similar to other surveys (*13*,*14*). Third, estimated R_0_ depends on the serial interval. Because no data from a local study of infector–infectee pairs were available, the distribution of the serial interval was based on previous estimates (*10*,*11*; D. Cereda et al., unpub. data, https://arxiv.org/abs/2003.09320). The estimated R_0_ aligned with estimates obtained in China (*44*) and Italy (*45*), and we accounted for the uncertainty in this value. We also repeated the analysis assuming a shorter serial interval (*12*), which resulted in a lower reproduction number. Fourth, in assessing the effect of each social distancing measure separately, we should note that an interrelation exists between the different measures and our approach might be an approximation. For example, school closure alone might result in increases in leisure contacts or decline in work contacts because parents need to be home with younger children. Fifth, as elsewhere, we assumed that changes in social contacts occur as soon as interventions take place, rather than gradually during lockdown dates (*28*), which could be valid for some interventions, such as school closure, but not for others. Finally, we did not consider case-based interventions that might have affected contacts, such as isolation of confirmed cases and quarantine of close contacts. In Greece, narrow testing criteria were applied beginning March 16 and elderly or severely ill persons, other high-risk groups, and healthcare personnel were tested but others were not; also, the testing capacity during March and April was low.

Overall, the social distancing measures Greece put in place in early March 2020 had a substantial impact on contact patterns and reduced R_0_ to <1.0. By the end of April, the spread of COVID-19 was contained in Greece, and the country had one of the lowest ARs in Europe after the first pandemic wave. However, as social distancing and travel restrictions are relaxed, close monitoring of R_t_ is essential in order to adapt interventions over time without having to resort to stringent measures. Measuring social mixing patterns and adherence to infection control measures through repeated surveys can be additional tools for real-time monitoring of the epidemic potential in the months to come.

Appendix Additional information and formulas used to calculate effects of social distancing measures during the first epidemic wave of severe acute respiratory syndrome coronavirus 2, Greece.
