# Supplementary material for: Effects of Social Distancing Measures during the First Epidemic Wave of Severe Acute Respiratory Syndrome Infection, Greece
Source: Emerg Infect Dis. 2021 Feb;27(2):452–62. doi: 10.3201/eid2702.203412 (PMC7853557; doi:10.3201/eid2702.203412)
Supplement: Appendix — Additional information and formulas used to calculate effects of social distancing measures during the first epidemic wave of severe acute respiratory syndrome coronavirus 2, Greece. [file 20-3412-Techapp-s1.pdf]

Article DOI: https://doi.org/10.3201/eid2702.203412

Effects of Social Distancing Measures during the First Epidemic Wave of Severe Acute Respiratory Syndrome Infection, Greece

Appendix

Social Contacts Survey

During March 31–April 7, 2020, we conducted a survey of social contacts among persons in Athens, Greece. Proportional quota sampling based on age and sex was used and persons 0–17 years old were oversampled. Random digital dialing was used to reach the population and only 1 person in each household was asked to participate to the study. Trained staff administered questionnaires by telephone. Calls were placed between 10:00 AM–3:00 PM and 5:30 PM–9:30 PM (Appendix Figure 1).

Eligible participants had to be local resident of Athens, and to have lived ≥6 months in Athens during the past year, which was applicable only for respondents >2 years of age. Time and budget restrictions did not allow expansion of the survey outside Athens. However, Athens Metropolitan Area includes 3.83 million of the 10.8 million persons residing in Greece.

The questionnaire consisted of three sections: 1) general information, such as sex, age, educational level, household size, and age of household members; 2) a contact diary for a 24-hour period from 5:00 AM of the day before the interview to 5:00 AM the day of the interview or the previous Friday if the interview took place on Monday; and 3) a contact diary for the same day of the week in mid-January before the first cases were diagnosed in Europe.

Parental-proxy completion was used for all children 0–11 years of age and for children and adolescents 12–17 years of age if the parent did not consent to provide information on their own. More specifically, interviews of persons <18 years old were performed as follows: parents or guardians responded to the questionnaire on behalf of children 0–11 years old; for children

and adolescents 12–17 years of age, either the participant provided information on their own with parental informed consent, or parents provided information on behalf of the participant. For parental-proxy completion, parents were asked to collaborate with their child if the child was old enough to provide information.

In social contacts studies, children often are deliberately oversampled because of their important role in the spread of infectious diseases (*1*,*2*). In our survey, we oversampled children and adolescents 0–17 years of age because we wanted to be able to assess social contacts in various age groups (0–4, 5–11, 12–17) and to explore the impact of school closure.

Estimates Assuming a Shorter Serial Interval

We also estimated R_0 assuming a shorter serial interval with mean of 4.7 days and standard deviation of 2.9 days (*3*). Using a shorter serial interval, estimated R_0 was 1.85 (95% CI 1.56–2.17) compared with an estimated R_0 of 2.38 (95% CI 2.01–2.80) under a longer serial interval in the main analysis. In the susceptible-exposed-infectious-recovered (SEIR) model, we assumed a duration of infectiousness of 3 days rather than 4.5 days under a longer serial interval (Appendix Figure 4).

Assuming a shorter serial interval, estimated effective reproduction number (R_t) was close to 1 before the implementation of lockdown. Then during lockdown, R_t was 0.35 (95% CrI 0.27–0.44) assuming a serial interval of 4.7 days compared with 0.46 (95% CrI 0.35–0.57) under a serial interval of 6.67 days. The cumulative number of infections from the start of the epidemic until the end of the simulations period on April 26, was 12,423 (95% CrI 5,562–28,713) compared with 13,189 infections (95% CrI 6,206–27,700) under a longer serial interval. The cumulative number of infections corresponds to an AR of 0.11% (95% CrI 0.05%–0.27%) compared with 0.12% (95% CrI 0.06%–0.26%) assuming the longer serial interval. At the end of the simulations period, April 26, the median number of new infections per day was predicted to reach 2.5 (95% CrI 0.5–14.4) compared with 25 new infections per day (95% CrI 6–97) with the longer serial interval. On April 26, the median number of infectious cases in our model was 22 (95% CrI 5–101) compared with 329 infectious cases (95% CrI 97–1,027) assuming a longer serial interval.

Based on the number of deaths reported in Greece by April 26, we estimated the infection fatality ratio (IFR) by using the number of infections as denominator with a time lag of 18 days. Using this calculation, estimated IFR was 1.11% (95% CrI 0.49%–2.47%) compared with 1.12% (95% CrI 0.55%–2.31%) assuming a longer serial interval.

Assessing the Impact of Social Distancing Measures

Estimating the Relative Change in R_0 before and during Social Distancing Measures

The relative change (δ) in R_0 before and during social distancing measures is equivalent to the reduction in the dominant eigenvalue of the contact matrices obtained for the 2 periods and was calculated as follows (equation [1]):

$$\delta = 1 - \frac{\max eigenvalue\ (K_{during})}{\max eigenvalue\ (K_{pre})}$$

where the elements of the matrices K_{pre} and K_{during} are defined as $k_{ij,pre} = s_i c_{ij,pre}$ and $k_{ij,during} = s_i c_{ij,during}$ in which $c_{ij,pre}$ is the average number of contacts between persons in age group *i* with persons in age group *j* before the period of social distancing measures, $c_{ij,during}$ is the number of contacts during the period of social distancing measures, and s_i is the susceptibility to infection of an age-*i* person (i,j = 1,..6).

Estimating the Social Contacts Matrix during the Initial Measures

The first period of measures during March 11–22, included closure of schools, entertainment venues, and shops except from supermarkets, grocery stores, and pharmacies. Because we did not measure the reduction in contacts during this period, we used the information from the contacts reported on a regular weekday in January 2020 and mimicked the impact of these intervention by excluding school contacts and reducing contacts at work and leisure activities accordingly (*2*,*4*–*6*). Thus, we created a synthetic contact matrix by assuming that no school contacts took place because of school closures and that contact through leisure activities was reduced by 80% and work contacts reduced by 30% during lockdown as a result of these first measures. We accounted for a reduction in work contacts because a special purpose leave was provided to working parents with children enrolled in nursery schools and kindergarten or with children $\leq$15 years of age attending mandatory education schools. Contacts reported at multiple locations, such as contact with a person at school and leisure activities, were assigned to

a single location using the following hierarchical order: home, work, school, leisure activities, transportation, and other locations (*4*). Thus, the social contacts matrix for the first period of measures was as follows:

$$C_{inital\ measures} = C_{home} + (1 - f_1) \cdot C_{work} + 0 \cdot C_{school} + (1 - f_2) \cdot C_{leisure} + C_{transport} + C_{other}$$

where $C_{home}, C_{work}, C_{school}, C_{leisure}, C_{transport}, C_{other}$ were the matrices obtained from the contacts reported on a regular weekday before the pandemic in Greece in January 2020 and f_1, f_2 are the reduction in leisure and work contacts during the first measures.

The relative change in R_0 was then estimated from equation [1] by using the contacts c_{ij} from the corresponding social contacts matrices $C_{inital\ measures}$ and C_{pre}.

Effects of Each Measure Implemented during Lockdown

To assess the impact of each measure separately, we estimated the reduction in R_0 by using the social contacts matrix before the pandemic and the synthetic matrix corresponding to each measure or combination of measures. For example, to estimate the impact of school closures, we compared the original matrix with social contacts reported on a regular weekday (C_{pre}) to the matrix resulting from the sum of home, work, leisure, transportation, and other contacts, excluding contacts in the school setting. The resulting synthetic contact matrix for school closure became

$$C_{school\ closure} = C_{home} + C_{work} + 0 \cdot C_{school} + C_{leisure} + C_{transportation} + C_{other}$$

Similarly, the impact of closing restaurants, coffee shops, cinemas, and other venues was estimated by reducing the subset of leisure contacts data by a proportion f. The synthetic contact matrix became

$$C_{reduction\ leisure} = C_{home} + C_{work} + C_{school} + (1 - f) \cdot C_{leisure} + C_{transportation} + C_{other}$$

This approach was used to assess the impact of a combination of measures, for example school closure and reduction in contacts at work, because they were measured during lockdown.

Effects of Milder Measures in Reducing Transmission during the First Wave

We assessed the impact of a theoretical scenario with less disruptive social distancing measures. A reduction of 50% in school contacts, such as classes split in half, combined with 20% teleworking and 20% reduction in leisure activities, results in the following contact matrix:

$$C_{mild\,measures} = C_{home} + (1-0.20)\cdot C_{work} + (1-0.50)\cdot C_{school} + (1-0.20)\cdot C_{leisure} + C_{transportation} + C_{other}$$

Effects of Lifting Measures Post Lockdown for Varying Effectiveness of Infection Control Measures

We assessed scenarios in which lockdown measures were partially lifted. As a result, the number of contacts increase but they do not return to the pre-epidemic levels. We hypothesized a scenario (scenario 1) in which contacts at work and school would return to levels that are 50% lower than prepandemic levels and leisure activities are 60% lower. The rational for this scenario is based on the following selected measures implemented post lockdown in Greece:

• High schools opened in mid-May and primary schools opened in June. Class sizes were reduced by half with a maximum of 15 students per classroom, desks were spaced 1.5 meters apart, and breaks were staggered to allow for physical distancing.

• Retail stores opened on May 11 with restrictions on the number of persons per square meter.

• Cafes, restaurants, and bars opened on June 1 with only outdoor spaces and restrictions on the number of people allowed per table.

Apart from scenario 1 (work reduced 50%, school 50%, leisure activities 60%.), we also assessed 2 scenarios with milder social distancing measures concerning the number of contacts post lockdown: scenario 2 involved work and leisure activities reduced by 20%, school by 50% and scenario 3 involved having all contacts are near prepandemic levels with just 20% reduction.

In each scenario, we applied the following methodology. The corresponding social contacts matrix for the period after lockdown is denoted as C_{post} and C_{during} is the contact matrix during lockdown. The resulting increase in R_t can be assessed as follows:

$$1-\frac{\max eigenvalue\ (K_{post})}{\max eigenvalue\ \ (K_{during})}$$

where the elements of the matrices K_{post} and K_{during} are defined as $k_{ij,post} = hs_i c_{ij,post}$ and $k_{ij,during} = s_i c_{ij,during}$ in which $c_{ij,post}$ and $c_{ij,during}$ are the average number of contacts between persons in age group *i* with persons in age group *j* post and during lockdown, and s_i is the susceptibility to infection of an age *i* person (*i*,*j* = 1,..6). We assumed that post lockdown

susceptibility to infection is reduced by a fraction (1 – h) as a result of intensive infection control measures, including hand hygiene, use of facemasks, and maintaining distances $\geq$1.5 m. We assumed the same reduction for all age groups.

We did not account for infection control measures during lockdown because contacts during that period occurred mostly within households. In addition, some measures, such as the use of fabric facemasks by the general public, were not recommended during lockdown in Greece. During the period of lifting lockdown measures, public health officials strongly recommended use of fabric facemasks by the general public and government officials made use of facemasks mandatory on public transportation and in crowded public spaces. To account for the efficacy of measures, such as keeping distances, and the possible impact of others, such as use of masks (*7*,*8*), we assumed a 5%–30% reduction in susceptibility (i.e., *h* ranging between 0.70–0.95) (Figure 6). This reduction corresponds to the efficacy and the adherence to these measures.

Under the scenarios with the milder social distancing measures (work and leisure contacts return to levels 20% below pre-epidemic, and school contacts are 20%–50% lower than pre-epidemic levels), infection control measures would need to reduce susceptibility to infection by 45%–50% (i.e., higher efficacy and adherence would be needed) (Appendix Figure 2).

Susceptible-Exposed-Infectious-Recovered (SEIR) Model

According to the model, susceptible persons (S) become infected at a rate, β, and move to the exposed state (E). At this point they are infected but not infectious. Exposed persons become infectious at a rate, σ, and a proportion, *p*, and will eventually develop symptoms. To account for asymptomatic transmission during the incubation period, we introduced a compartment for infectious cases who have not developed symptoms yet (I_{pre}). These persons develop symptoms at a rate, σ_s (I_{symp}). The remainder (1 – *p*) will be true asymptomatic or subclinical cases (I_{asymp}). We assumed that the infectiousness of these subclinical cases relative to symptomatic is *q*. Symptomatic cases recover (R) at a rate, γ_s, and asymptomatic at a rate, γ_{asymp}. Only cases in compartments I_{pre}, I_{symp}, and I_{asymp} are assumed to be infectious. The transitions between the compartments of the model are described by the following set of equations:

Susceptible, $\frac{dS}{dt} = -\delta_t \cdot \beta \cdot \left(q \cdot I_{asymp} + I_{pre} + I_{symp}\right) \cdot \frac{S}{N}$

Exposed, $\frac{dE}{dt} = \delta_t \cdot \beta \cdot \left(q \cdot I_{asymp} + I_{pre} + I_{symp}\right) \cdot \frac{S}{N} - \sigma \cdot E + \text{import}$

Infectious before developing symptoms, $\frac{dI_{pre}}{dt} = p \cdot E \cdot \sigma - \sigma_s \cdot I_{pre}$

Infectious and symptomatic, $\frac{dI_{symp}}{dt} = \sigma_s \cdot I_{pre} - \gamma_s \cdot I_{symp}$

Infectious and true asymptomatic (subclinical cases), $\frac{dI_{asymp}}{dt} = (1-p) \cdot E \cdot \sigma - \gamma_{asymp} \cdot I_{asymp}$

Recovered, $\frac{dR}{dt} = \gamma_s \cdot I_{symp} + \gamma_{asymp} \cdot I_{asymp}$

The parameter β is estimated through R_0 from the following equation:

$$\beta = \frac{R_0}{(1-p)q\frac{1}{\gamma_{asymp}} + p(\frac{1}{\sigma_s} + \frac{1}{\gamma_s})}$$

To incorporate the impact of social distancing in the model, the infection rate β was multiplied by the parameter δ_t (t = 1,2) corresponding to the reduction of R_0 in 2 periods of social distancing measures. We considered 2 major periods of social distancing measures: March 11–22, the period of initial measures including closure of schools, restaurants, shopping centers, cinemas, etc. until the day before lockdown, and March 23–April 27, the period of lockdown. Based on the social contacts data, we estimated not only the reduction in the total number of contacts but also in the number of contacts at work, home, school, and leisure activities during lockdown. Thus, we modeled the relative reduction in R_0 in the 2 periods of social distancing measures, as described in the manuscript and Appendix.

We assumed that local transmission initiated on February 15, 2020 because the earliest reported date of symptom onset among locally infected cases was February 20. In our model, we seeded 1 symptomatic case in the population at day 0 (February 15th) and further seeded the epidemic by 700 imported cases over the first 40 days. This assumption was based on the ≈500

imported cases diagnosed by April 7 in Greece and taking into account unreported asymptomatic imported cases (*9*).

As a sensitivity analysis, we obtained model predictions assuming that asymptomatic and symptomatic persons are equally infectious (q = 100%) and the results were similar. For example, the cumulative number of infections from the start of the epidemic until the end of the simulation period was 13,066 (95% CrI 6,012–27,112) assuming q = 100% compared with 13,189 infections (95% CrI 6,206–27,700) assuming q = 50%.

Infection Fatality Ratio (IFR) and Comparison of Observed Deaths to Model Predictions

To validate our findings, we used a reverse approach; we applied a published estimate of the IFR (*10*) to the number of infections predicted by the model and compared the resulting number of deaths (cumulative and daily number) to the observed.

We adjusted the IFR estimate by Verity et al (*10*) to account for nonhomogeneous attack rates across age groups, as proposed elsewhere (*11*), and for the age distribution of the population of Greece. To account for the lower ARs among younger persons (12–14 years of age), we multiplied the age-specific IFR for persons 0–9 and 10–19 years of age by 1/0.34, where 0.34 is the relative susceptibility to infection of these age groups compared to adults (*12*). The corrected age specific IFRs were then combined to produce an overall IFR adjusting for the age distribution of the population in Greece (Appendix Table 3). To validate the model, we applied this IFR to the total number of infections predicted by the model and assumed a lag of 18 days between infection and death to compare the predicted number of deaths to the cumulative number of reported deaths (Appendix Figure 2).

References

1. Mossong J, Hens N, Jit M, Beutels P, Auranen K, Mikolajczyk R, et al. Social contacts and mixing patterns relevant to the spread of infectious diseases. PLoS Med. 2008;5:e74. PubMed https://doi.org/10.1371/journal.pmed.0050074

2. Hens N, Ayele GM, Goeyvaerts N, Aerts M, Mossong J, Edmunds JW, et al. Estimating the impact of school closure on social mixing behaviour and the transmission of close contact infections in

eight European countries. BMC Infect Dis. 2009;9:187. PubMed https://doi.org/10.1186/1471-2334-9-187

3. Nishiura H, Linton NM, Akhmetzhanov AR. Serial interval of novel coronavirus (COVID-19) infections. Int J Infect Dis. 2020;93:284–6. PubMed https://doi.org/10.1016/j.ijid.2020.02.060

4. Willem L, Van Hoang T, Funk S, Coletti P, Beutels P, Hens N. SOCRATES: an online tool leveraging a social contact data sharing initiative to assess mitigation strategies for COVID-19. BMC Rese Notes. 2020;13:293. PubMed https://doi.org/10.1186/s13104-020-05136-9

5. Prem K, Liu Y, Russell TW, Kucharski AJ, Eggo RM, Davies N, et al.; Centre for the Mathematical Modelling of Infectious Diseases COVID-19 Working Group. The effect of control strategies to reduce social mixing on outcomes of the COVID-19 epidemic in Wuhan, China: a modelling study. Lancet Public Health. 2020;5:e261–70. PubMed https://doi.org/10.1016/S2468-2667(20)30073-6

6. Davies NG, Kucharski AJ, Eggo RM, Gimma A, Edmunds WJ, Jombart T, et al.; Centre for the Mathematical Modelling of Infectious Diseases COVID-19 working group. Effects of non-pharmaceutical interventions on COVID-19 cases, deaths, and demand for hospital services in the UK: a modelling study. Lancet Public Health. 2020;5:e375–85. PubMed https://doi.org/10.1016/S2468-2667(20)30133-X

7. Leung NHL, Chu DKW, Shiu EYC, Chan KH, McDevitt JJ, Hau BJP, et al. Respiratory virus shedding in exhaled breath and efficacy of face masks. Nat Med. 2020;26:676–80. PubMed https://doi.org/10.1038/s41591-020-0843-2

8. National Academies of Sciences, Engineering, and Medicine. Rapid expert consultation on the effectiveness of fabric masks for the COVID-19 pandemic; April 8, 2020. Washington, DC: The National Academies Press;2020. https://doi.org/10.17226/25776

9. National Public Health Organisation. Epidemiological surveillance of COVID-19– daily report 26 April 2020 [in Greek] [cited 2020 Jul 10]. https://eody.gov.gr/wp-content/uploads/2020/04/covid-gr-daily-report-20200426.pdf

10. Verity R, Okell LC, Dorigatti I, Winskill P, Whittaker C, Imai N, et al. Estimates of the severity of coronavirus disease 2019: a model-based analysis. Lancet Infect Dis. 2020;20:669–77. PubMed https://doi.org/10.1016/S1473-3099(20)30243-7

11. Flaxman S, Mishra S, Gandy A, Unwin HJT, Mellan TA, Coupland H, et al.; Imperial College COVID-19 Response Team. Estimating the effects of non-pharmaceutical interventions on

COVID-19 in Europe. Nature. 2020;584:257–61. PubMed https://doi.org/10.1038/s41586-020-2405-7

12. Zhang J, Litvinova M, Liang Y, Wang Y, Wang W, Zhao S, et al. Changes in contact patterns shape the dynamics of the COVID-19 outbreak in China. Science. 2020;368:1481–6. PubMed https://doi.org/10.1126/science.abb8001

13. Jing QL, Liu MJ, Zhang ZB, Fang LQ, Yuan J, Zhang AR, et al. Household secondary attack rate of COVID-19 and associated determinants in Guangzhou, China: a retrospective cohort study. Lancet Infect Dis. 2020;20:1141–50. PubMed https://doi.org/10.1016/S1473-3099(20)30471-0

14. Li W, Zhang B, Lu J, Liu S, Chang Z, Peng C, et al. The characteristics of household transmission of COVID-19. Clin Infect Dis. 2020;71:1943–6. PubMed https://doi.org/10.1093/cid/ciaa450

15. European Centre for Disease Prevention and Control. Daily number of new reported cases of COVID-19 by country worldwide 2020 May 20 [cited 2020 May 20]. https://www.ecdc.europa.eu/en/publications-data/download-todays-data-geographic-distribution-covid-19-cases-worldwide

Appendix Table 1. Main control measures implemented in Greece during the coronavirus disease pandemic, February 26–March 29, 2020

Start date, 2020	Description
Feb 26	Testing and isolation of confirmed or suspected cases and their contacts
Feb 27	Ban of carnival festivities
Mar 5	Testing and isolation of confirmed or suspected cases and their contacts in outbreaks and superspreading events
Mar 9	Ban of flights to northern Italy
Mar 9	Suspension of open care centers and cancellation of indoor conferences and sporting events
Mar 10	Ban of outdoor mass gatherings and sporting events
Mar 11	School and university closures
Mar 13	Closure of all theatres, cinemas, gyms, playgrounds, clubs, and courthouses
Mar 14	Ban of flights to Italy
Mar 14	Closure of shopping centers, archeological sites, bars, and restaurants
Mar 15	Border closure to Albania and North Macedonia
Mar 16	Ban of religious services
Mar 18	Border closure to non-European Union nationals
Mar 18	Nationwide closure of all private enterprises
Mar 19	Closure of sea borders
Mar 20	14-day quarantine for inbound travelers
Mar 23	Border closure to United Kingdom and Turkey
Mar 23	Ban of all intra- and inter-city movements across country (complete lock down)
Mar 23	Hotel closures
Mar 26	Testing of inbound travelers from countries with high rate of transmission
Mar 29	Border closure to the Netherlands and Germany

Appendix Table 2. Literature estimates concerning the relative susceptibility to severe acute respiratory syndrome coronavirus 2 infection by age*

Reference and data	Relative susceptibility to infection, odds ratio (95% CI)
Zhang et al. (*12*); 7,375 contacts from 114 clusters; age, y	
0–14	0.34 (0.24–0.49)
15–64	Referent
>65	1.47 (1.12–1.92)
Jing et al. (*13*); 2,075 contacts of 212 primary cases in 195 unrelated clusters; age, y	
0–19	0.27 (0.13–0.55)
20–59	0.80 (0.53–1.19)
>60	Referent
Li et al. (*14*); 392 household contacts of 105 index patients; age, y	
0–17	0.18 (0.06, 0.54)
>18	Referent

*Although reference *12* and *13* do not use exactly the same age categories, we note that in Jing et al. (*13*), the odds ratio of infection for persons 0–19 years of age versus persons 20–59 years of age is 0.34 (0.27/0.80), which is similar to that estimated by Zhang et al. (*12*) for the comparison of persons 0–14 years of age vs. persons 15–64 years of age.

Appendix Table 3. Infection fatality ratio adjusted for the age distribution of the population and relative susceptibility to infection by age, Greece*

Age group, y	No. Greece	IFR, % (*10*)	Relative susceptibility†	Adjusted IFR, %‡	IFR standardized for the age distribution of the population in Greece, %§
0–9	1,049,839	0.00161	0.34	0.00474	1.14 (0.62–2.19)
10–19	1,072,705	0.00695	0.34	0.02044	
20–29	1,350,868	0.0309	1	0.0309	
30–39	1,635,304	0.0844	1	0.0844	
40–49	1,581,095	0.161	1	0.161	
50–59	1,391,854	0.595	1	0.595	
60–69	1,134,045	1.93	1	1.93	
70–79	1,017,242	4.28	1	4.28	
>80	583,334	7.80	1	7.80	

*IFR is based on published estimates from Verity et al. (*10*). IFR, infection fatality ratio.
†Relative susceptibility to infection based on Zhang et al. (*12*).
‡IFR adjusted for age susceptibility.
§The lower and upper limits were calculated using the upper and lower bounds of the age specific IFR provided by Verity et al. (*10*).

Appendix Table 4. Number of coronavirus disease deaths per million population in Europe by May 18, 2020*

Country or territory	Population, 2018	Total deaths	Deaths/million population
San Marino	33,785	41	1,213.56
Belgium	11,422,068	9,052	792.5
Andorra	77,006	51	662.29
Italy	60,431,283	31,908	528
United Kingdom	66,488,991	34,636	520.93
France	66,987,244	28,108	419.6
Sweden	10,183,175	3,679	361.28
The Netherlands	17,231,017	5,680	329.64
Ireland	4,853,506	1,543	317.91
Isle of Man	84,077	24	285.45
Jersey	106,800	27	252.81
Guernsey	63,026	13	206.26
Switzerland	8,516,543	1,602	188.1
Luxembourg	607,728	107	176.07
Monaco	38,682	5	129.26
Portugal	10,281,762	1,218	118.46
Germany	82,927,922	7,935	95.69
Denmark	5,797,446	547	94.35
Austria	8,847,037	629	71.1
Moldova	3,545,883	211	59.51
Romania	19,473,936	1,097	56.33
Finland	5,518,050	298	54
Slovenia	2,067,372	104	50.31
North Macedonia	2,082,958	101	48.49
Estonia	1,320,884	63	47.7
Hungary	9,768,785	462	47.29
Norway	5,314,336	232	43.66
Bosnia and Herzegovina	3,323,929	132	39.71
Serbia	6,982,084	230	32.94
Iceland	353,574	10	28.28
Czechia	10,625,695	298	28.05
Liechtenstein	37,910	1	26.38
Poland	37,978,548	925	24.36
Croatia	4,089,400	95	23.23
Armenia	2,951,776	60	20.33
Lithuania	2,789,533	56	20.08
Russia	144,478,050	2,631	18.21
Belarus	9,485,386	165	17.4
Kosovo	1,845,300	29	15.72
Bulgaria	7,024,216	110	15.66
**Greece**	**10,727,668**	**163**	**15.19**
Montenegro	622,345	9	14.46
Cyprus	1,189,265	17	14.29
Malta	483,530	6	12.41
Ukraine	44,622,516	514	11.52
Albania	2,866,376	31	10.82
Latvia	1,926,542	19	9.86
Slovakia	5,447,011	28	5.14
Azerbaijan	9,942,334	39	3.92
Georgia	3,731,000	12	3.22
Faroe Islands	48,497	0	0
Gibraltar	33,718	0	0
Holy See	1,000	0	0

*Using data from European Centre for Disease Prevention and Control (*15*). Countries are listed from highest to lowest death rate. Bold text indicates Greece's lower death rate compared with other countries.

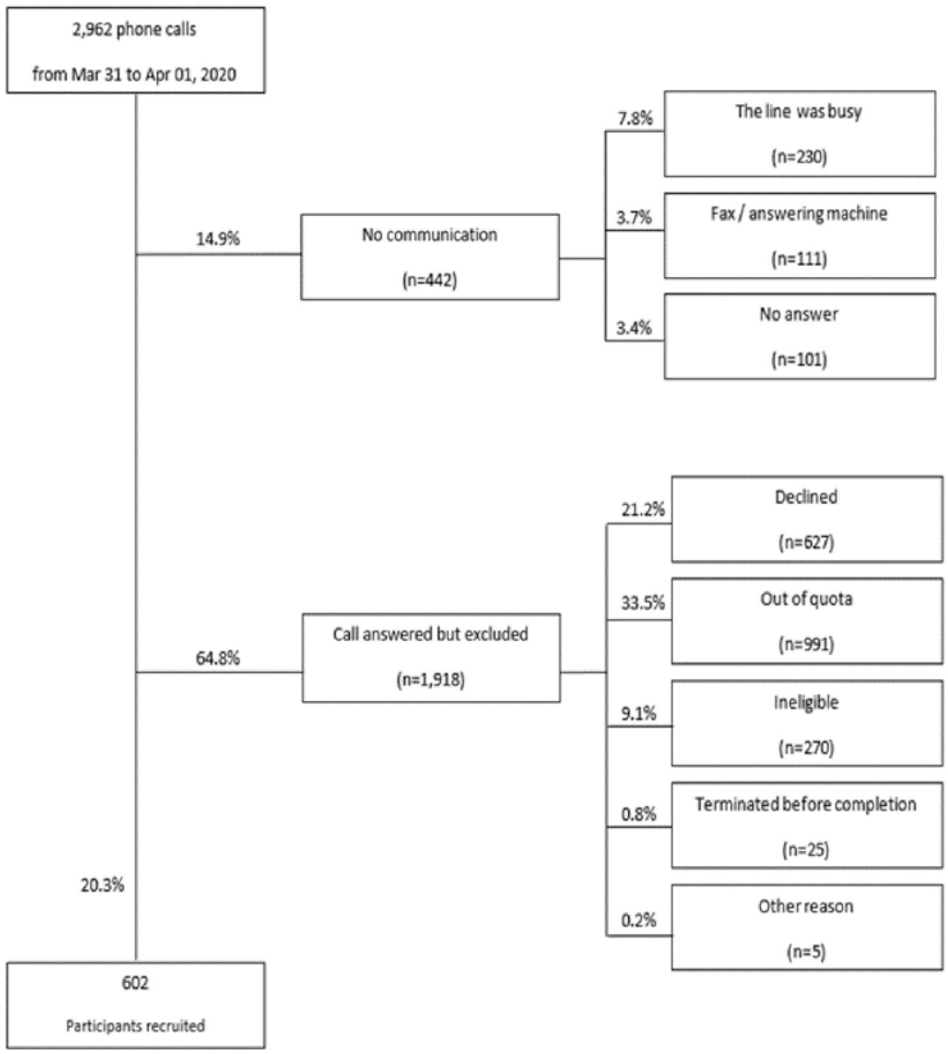

Appendix Figure 1. Flow chart of the recruitment process for a social contacts survey used to assess effects of social distancing measures during the first epidemic wave of severe acute respiratory syndrome coronavirus 2, Greece.

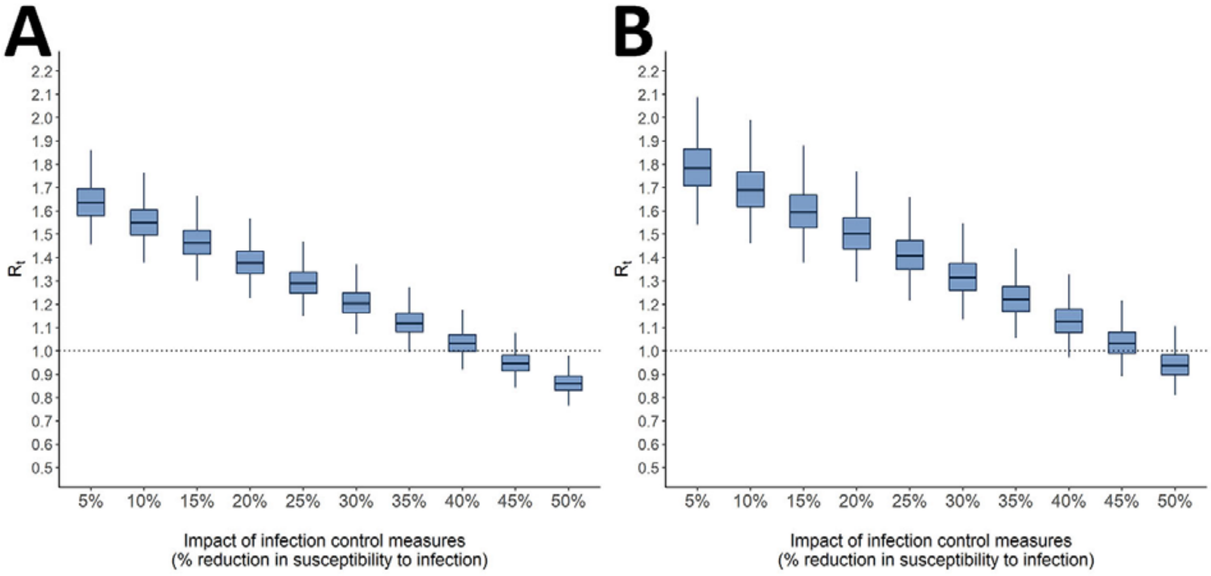

Appendix Figure 2. Estimated R_t after the partial lifting of social distancing measures at the end of the first SARS-CoV-2 wave in Greece. We assumed varying effectiveness levels of infection control measures (e.g., hand hygiene, use of masks, keeping distances) in reducing susceptibility to infection. R_t during lockdown was 0.46. For the partial lifting of measures, we hypothesized 2 additional scenarios: A) Contacts at work, return to levels that are 20%, school to 50%, and leisure activities to 20% below pre-epidemic levels; and B) contacts at work, school, and leisure activities all return to levels that are 20% below pre-epidemic levels. R_t, effective reproduction number; SARS-CoV-2, severe acute respiratory syndrome coronavirus 2.

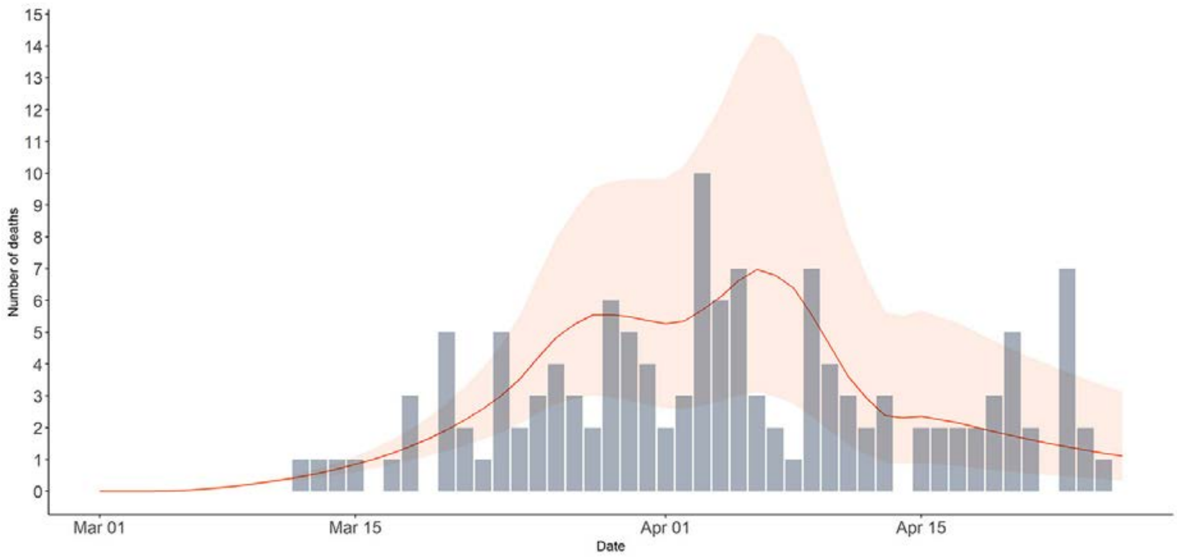

Appendix Figure 3. Observed number of deaths per day compared with model estimates for the first epidemic wave of SARS-CoV-2, Greece. Gray bars indicate observed number of deaths; orange line indicates median; and orange shading indicates 95% CrI range of the model estimates. We used a published estimate of the infection fatality ratio (IFR) (*10*) adjusted for nonhomogenous attack rates by age and for the age distribution of the population of Greece (IFR = 1.14%; Appendix Table 3). The estimated number of deaths was obtained by applying this IFR to the total number of infections predicted by the model assuming a delay of 18 days from infection to death. Locally weighted smoothing was applied to the model estimates in the graph. The observed number of deaths was obtained from epidemiological surveillance data (*9*). SARS-CoV-2, severe acute respiratory syndrome coronavirus 2.

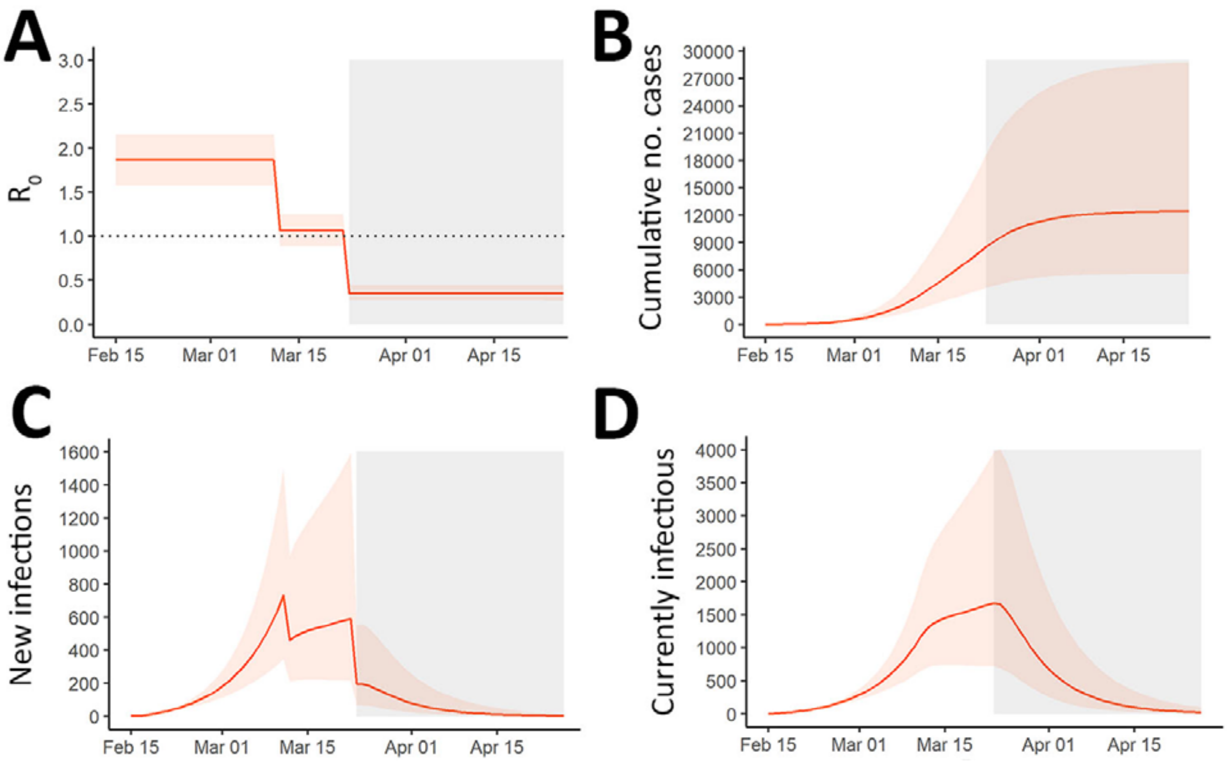

Appendix Figure 4. Estimates for the first wave of the SARS-CoV-2 epidemic assuming a shorter serial interval (mean 4.7 days) in a SEIR model for Greece, February 15–April 26, 2020. Model estimates for A) Effective reproduction number; B) cumulative number of cases; C) number of new infections; and D) current number of infected persons. Solid orange line represents median estimates; light orange shaded areas indicate 95% credible intervals from 1,000 simulations of the SEIR model. Gray zone indicates estimates during lockdown, the period in which all nonessential movement in the country was restricted. SARS-CoV-2, severe acute respiratory syndrome coronavirus 2; SEIR, susceptible-exposed-infected-recovered.
